# Episodic Ataxia Type 2 Presenting with Fluctuating Weakness in a Child with a De Novo CACNA1A Variant

**DOI:** 10.3390/children13040488

**Published:** 2026-03-31

**Authors:** Sungyeon Park, Hyunwoo Bae, Soonhak Kwon, Yun Jeong Lee

**Affiliations:** 1 School of Medicine, Kyungpook National University, Daegu 41944, Republic of Korea; angelysugar18@knu.ac.kr; 2 Department of Pediatrics, Kyungpook National University Children’s Hospital, School of Medicine, Kyungpook National University, Daegu 41944, Republic of Korea; hbae@knu.ac.kr (H.B.); shkwon@knu.ac.kr (S.K.); 3 Department of Pediatrics, Kyungpook National University Hospital, School of Medicine, Kyungpook National University, Daegu 41944, Republic of Korea

**Keywords:** episodic ataxia type 2, CACNA1A, cognition, muscle weakness

## Abstract

Background: Episodic ataxia type 2 (EA2) is the most common subtype of episodic ataxia and is primarily caused by pathogenic variants in the *CACNA1A* gene. Although classically characterized by paroxysmal ataxia, *CACNA1A*-related disorders are increasingly recognized as an age-dependent phenotypic continuum that extends beyond episodic cerebellar dysfunction to include fluctuating weakness, persistent neurological signs, and neurodevelopmental impairments. Case report: A 12-year-old boy presented with episodic vertigo. His medical history was notable for infantile paroxysmal tonic upward gaze beginning at 6 months of age. From the age of 7 years, he developed frequent episodes of vertigo and ataxia lasting 2 to 3 h. At 10 years of age, he experienced an episode of acute lower limb weakness with diminished deep tendon reflexes, without prominent ataxia. Guillain–Barré syndrome was initially suspected, and he received two courses of intravenous immunoglobulin, with only transient improvement. Neurophysiological studies were largely unremarkable, except for an isolated decremental response on repetitive nerve stimulation. In addition to paroxysmal events, he exhibited persistent interictal cerebellar signs, including dysmetria, dysdiadochokinesia, and downbeat nystagmus. Neuropsychological testing revealed mild intellectual disability with prominent visuospatial deficits. Trio-based whole-exome sequencing identified a de novo *CACNA1A* splice donor variant (c.978 + 1G > A), confirming the diagnosis of EA2. Treatment with acetazolamide resulted in marked improvement in episodic ataxic events. Conclusions: This case highlights EA2 as part of a broader *CACNA1A*-related phenotypic continuum rather than a purely paroxysmal disorder. Awareness of atypical and age-dependent manifestations is crucial to avoid diagnostic pitfalls and to facilitate the timely initiation of targeted therapy and appropriate developmental support.

## 1. Introduction

Episodic ataxia type 2 (EA2) is a rare autosomal dominant neurological disorder characterized by paroxysmal episodes of vertigo, ataxia and imbalance. Although EA2 is the most prevalent subtype of episodic ataxia, it remains a rare condition with an estimated incidence of less than 1 per 100,000 individuals [1]. It results from heterozygous pathogenic variants in *CACNA1A*, which encodes the α1A subunit of the P/Q-type calcium channel Cav2.1 [1]. Clinically, EA2 typically presents with recurrent episodes lasting hours to days. These attacks are frequently triggered by physiological or emotional stressors, such as physical exertion, caffeine, or alcohol consumption [2]. During attacks, patients may exhibit severe ataxia, dysarthria, and primary-position downbeat or gaze-evoked nystagmus. Approximately 90% of individuals with EA2 also exhibit interictal nystagmus, predominantly gaze-evoked or primary-position downbeat nystagmus [3].

Traditionally, *CACNA1A* pathogenic variants have been classified into three distinct allelic disorders based on their molecular mechanisms: loss-of-function variants causing EA2, gain-of-function missense pathogenic variants leading to familial hemiplegic migraine type 1 (FHM1), and CAG repeat expansions resulting in spinocerebellar ataxia type 6 (SCA6) [4]. However, this rigid genotype–phenotype distinction is evolving toward an integrated, age-dependent phenotypic continuum [4]. Clinical boundaries are increasingly blurred, as EA2 is no longer restricted to paroxysmal cerebellar dysfunction but is known to include intellectual disability, attention-deficit/hyperactivity disorder (ADHD), epilepsy, dystonia, migraine, varying degrees of drowsiness, confusion, or coma and fluctuating weakness [5,6,7]. Moreover, several studies have described persistent cerebellar signs or gradual disease progression in some individuals, suggesting phenotypic overlap with SCA6 [8,9]. Despite this progress, atypical presentations often lead to significant diagnostic challenges. Clinical features beyond episodic vertigo and ataxia have received little attention in discussions of EA2.

Herein, we describe a pediatric case of EA2 presenting with a complex history of infantile tonic upgaze, fluctuating muscle weakness, and cognitive impairment, reinforcing the necessity of a broad clinical perspective in *CACNA1A*-related disorders.

## 2. Case Report

**Figure 1:**
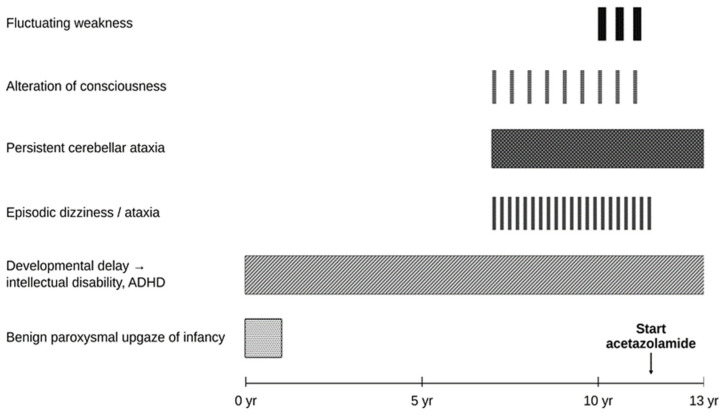
Clinical course of the patient showing episodic symptoms, diagnostic process, and treatment response over time.

A 12-year-old boy was referred to our pediatric neurology clinic for evaluation of episodic dizziness. His symptoms had begun at the age of seven years and were characterized by episodes of vertigo lasting approximately 2–3 h, occurring two to three times per week. During these episodes, the patient experienced gait disturbance and imbalance, which typically improved after sleep. On some occasions, the episodes were also accompanied by transient alterations in consciousness, described as drowsiness and reduced responsiveness. The clinical course, diagnostic process, and treatment response over time are summarized in Figure 1.

At the age of 10 years, the patient experienced an acute episode of lower limb weakness that progressed to difficulty maintaining independent standing. Neurological examination at that time revealed reduced motor strength in the lower extremities (Medical Research Council grade 3/5) and diminished deep tendon reflexes (DTRs). Notably, this episode occurred without prominent vertigo or ataxia. Guillain–Barré syndrome (GBS) was therefore initially suspected. Cerebrospinal fluid analysis revealed normal cell count, protein level, and glucose level, without albuminocytologic dissociation. Serum antiganglioside antibodies (including anti-GM1, GD1a, and GQ1b) were tested and were negative. The patient received intravenous immunoglobulin therapy and showed near-complete clinical recovery within approximately two weeks. Nerve conduction studies (NCS) performed approximately 10 days after the onset of weakness did not demonstrate findings consistent with demyelinating polyneuropathy.

Approximately one month later, at the age of 11 years, he experienced a second episode of generalized weakness accompanied by diffuse body pain, leading to a second course of intravenous immunoglobulin treatment. Subsequently, intermittent episodes of fluctuating weakness involving the upper extremities were reported. Because of the recurrent and fluctuating nature of the symptoms, additional neurophysiological studies were performed. NCS and needle electromyography (EMG) did not demonstrate findings consistent with inflammatory demyelinating polyneuropathy. A repetitive nerve stimulation (RNS) test was performed in the right orbicularis oculi, trapezius, abductor digiti minimi, and flexor carpi ulnaris muscles. A decremental response of approximately 15–17% was observed in the orbicularis oculi during 3 Hz stimulation, with a baseline CMAP amplitude of 1.04 mV. However, no consistent decrement was observed in the other tested muscles. These findings were considered insufficient to establish a definitive neuromuscular transmission disorder. Single-fiber EMG was not performed.

The patient was born at term following an uncomplicated pregnancy and delivery. Early development was mildly delayed. Independent walking was achieved at approximately fifteen months of age, and first meaningful words emerged around two years of age. At around six months of age, the patient developed paroxysmal tonic upward gaze of infancy, which persisted for approximately one year and resolved spontaneously.

At the time of evaluation in our clinic, neurological examination revealed persistent cerebellar signs, including dysmetria, dysdiadochokinesia, and an unsteady tandem gait. Oculomotor examination demonstrated primary-position downbeat nystagmus and hypometric saccades. Additionally, he showed decreased motor strength (MRC grade 4) in the left lower extremity and reduced DTRs.

Brain magnetic resonance imaging showed no structural abnormalities. Electroencephalography showed normal background activity during both wakefulness and sleep, without epileptiform discharges. Metabolic and autoimmune screening, including serum electrolytes, routine chemistry panels, metabolic screening for inherited metabolic disorders (tandem mass spectrometry, serum amino acid analysis, and urine organic acid analysis), antinuclear antibody, rheumatoid factor, and anti-double-stranded DNA antibody, were all within normal limits. Acetylcholine receptor antibody was negative, and creatine kinase was 102 U/L. Serum potassium, measured near the time of the weakness episode, was 4.1 mmol/L, effectively excluding hypokalemic periodic paralysis. Serial NCS and RNS tests showed no evidence of peripheral neuropathy or neuromuscular junction (NMJ) disorder. The previously observed decremental response in the orbicularis oculi was not reproduced on repeat testing, further arguing against a definitive NMJ disorder.

Neuropsychological assessment using the Korean version of the Wechsler Intelligence Scale for Children–Fifth Edition revealed a Full-Scale IQ score of 69. Index scores were as follows: verbal comprehension, 81; visuospatial, 62; fluid reasoning, 83; working memory, 89; and processing speed, 79. Assessments via the Swanson, Nolan, and Pelham Questionnaire (SNAP-IV) and the Comprehensive Attention Test confirmed symptoms consistent with ADHD, primarily the inattentive-subtype.

**Figure 2:**
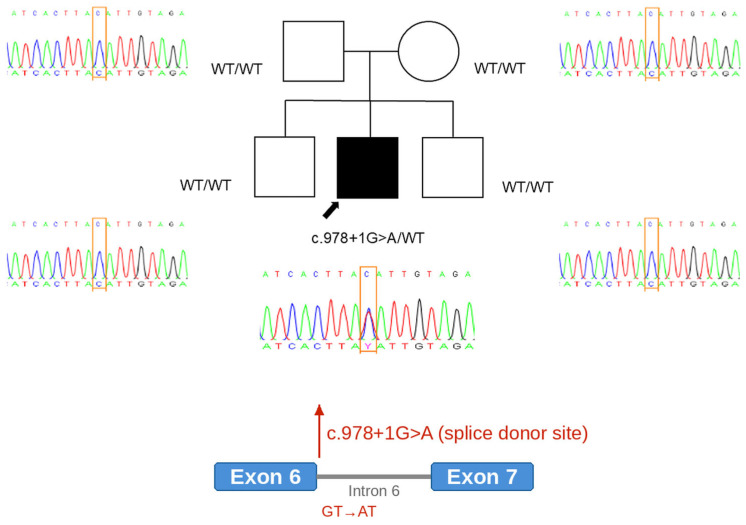
Pedigree of the patient and sequencing analysis demonstrating the presence of a heterozygous c.978 + 1G > A variant in the *CACNA1A* gene in the affected patient. WT/WT indicates homozygous wild-type at the c.978 + 1 position, confirmed by Sanger sequencing in all unaffected family members.

Given the persistent cerebellar dysfunction, we followed a stepwise genetic diagnostic approach. We first performed genetic testing for common spinocerebellar ataxias (SCA1, 2, 3, 6, 7, 8, and 17) to rule out CAG repeat expansions, which yielded negative results. Subsequently, trio-based whole-exome sequencing (Illumina NovaSeq platform) was performed with a mean depth of coverage of 153.3×, with 98% of bases meeting the quality threshold of ≥10×. It identified a heterozygous de novo splice donor variant in *CACNA1A* (c.978 + 1G > A; transcript NM_001127222.2), confirmed by Sanger sequencing (Figure 2). RNA studies were not performed, which precludes direct confirmation of aberrant splicing. According to ACMG/AMP criteria, the variant was classified as pathogenic based on PVS1 (canonical splice-site variant), PS2 (de novo occurrence), and PM2 (absence from population databases). Based on these findings, the patient was diagnosed with episodic ataxia type 2 (EA2). Acetazolamide therapy was initiated at a dose of 250 mg twice daily (approximately 10 mg/kg/day). Following treatment initiation, the episodic vertigo and ataxia showed near-complete resolution, and the fluctuating weakness episodes have not recurred during 15 months of follow-up. No adverse effects were noted, and renal function and serum electrolytes were monitored periodically and remained within normal limits throughout the follow-up period. The patient had no history of migraine or recurrent headaches.

## 3. Discussion

This case highlights the phenotypic diversity of EA2 and the diagnostic hurdles associated with its atypical manifestations. A significant diagnostic pitfall was the initial misdiagnosis of GBS. While GBS characteristically presents as a monophasic, progressive ascending paralysis, the fluctuating and recurrent nature of our patient’s weakness was fundamentally inconsistent with this diagnosis. Importantly, cerebrospinal fluid analysis showed normal cell counts and protein levels without albuminocytologic dissociation, serum antiganglioside antibodies were negative, and nerve conduction studies, performed approximately 10 days after symptom onset did not reveal demyelinating features—collectively arguing against a diagnosis of GBS. The broader differential for episodic weakness presenting without prominent ataxia also included periodic paralysis and other channelopathies, metabolic triggers, and the inflammatory neuropathy spectrum. Among these, a *CACNA1A*-related channelopathy most parsimoniously accounts for the recurrent, self-limited nature of the episodes, the absence of supportive evidence for inflammatory or metabolic etiologies, and the broader clinical context of interictal cerebellar signs and neurodevelopmental impairment.

Such fluctuating motor weakness is often underappreciated clinically, yet it has been increasingly documented as a manifestation of EA2, with a plausible mechanistic basis in impaired presynaptic Cav2.1 channel function at the NMJ [10,11]. At the NMJ, Cav2.1 channels serve as the primary mediators of calcium influx at the presynaptic terminal, directly triggering the exocytosis of acetylcholine vesicles [12]. *CACNA1A* loss-of-function pathogenic variants consequently diminish evoked acetylcholine release, compromising the safety factor of neuromuscular transmission [12]. Clinically, this presynaptic dysfunction often manifests as fluctuating muscle weakness that mimics the electrophysiological features of presynaptic disorders such as Lambert–Eaton myasthenic syndrome [10]. In our case, standard NCS and EMG were unremarkable. However, an approximately 15% decremental response was observed in the orbicularis oculi, while other muscles tested showed no significant decrement during the RNS test. This isolated finding falls at the conventional threshold of significance and is insufficient to confirm a definitive neuromuscular transmission defect. Single-fiber EMG, the most sensitive tool for detecting presynaptic dysfunction, was not performed in our patient, and this constitutes an important limitation. Definitive characterization of NMJ involvement would require confirmatory testing with single-fiber EMG in future cases, and interpretations of presynaptic failure based solely on borderline RNS findings should be made with caution.

Furthermore, this case emphasizes the significant neurodevelopmental burden of *CACNA1A* pathogenic variants. Emerging evidence suggests that global developmental delay, executive dysfunction, autism spectrum disorder, and learning disability are prevalent in this patient population [2,6,7]. Our patient’s early milestones were mildly delayed, with independent ambulation achieved at fifteen months and first meaningful words emerging at approximately two years of age. Subsequent neuropsychological assessment revealed persistent cognitive impairment, including a Full-Scale IQ of 69 and inattentive-subtype ADHD. The specific deficit in visuospatial processing observed in our patient is particularly characteristic of Cerebellar Cognitive Affective Syndrome (CCAS), a condition wherein cerebellar dysfunction manifests as impairments in executive function and spatial cognition [13]. Beyond cerebellar involvement, *CACNA1A* loss-of-function contributes to global synaptic abnormalities and impaired long-term potentiation in the hippocampus and prefrontal cortex [14], providing a mechanistic basis for the memory, attention, and executive function deficits observed in this patient. A recent study further demonstrated that the degree of CaV2.1 structural preservation correlates significantly with full-scale IQ and verbal comprehension in patients with EA2, suggesting that mutation-induced conformational disruption of the channel constitutes a molecular substrate for cognitive heterogeneity in this disorder [15]. These findings underscore the importance of routine neuropsychological evaluation and multidisciplinary neurodevelopmental support as integral components of EA2 management.

In addition to episodic weakness, our patient experienced transient alterations of consciousness, described as drowsiness and reduced responsiveness, accompanying some of the vertigo episodes. Electroencephalography demonstrated normal background activity during wakefulness and sleep, without epileptiform discharges, suggesting these events reflect ictal cerebellar or brainstem dysfunction rather than epileptic activity. This feature, though understated in many reports, is recognized within the *CACNA1A*-related phenotypic spectrum and warrants documentation when present [5].

In the present case, the patient exhibited not only episodic ataxic events but also persistent cerebellar signs, including ataxia, dysmetria, dysdiadochokinesia, and interictal nystagmus, which overlap significantly with the clinical phenotype of SCA6. In a cohort of 12 patients with *CACNA1A* haploinsufficiency, 75% were reported to develop progressive ataxia during adolescence or adulthood, and 88% exhibited interictal downbeat nystagmus [7]. The present patient will therefore require longitudinal monitoring for phenotypic progression toward the SCA6 spectrum.

Of particular interest is the patient’s history of paroxysmal tonic upward gaze during infancy, which persisted for approximately one year. Paroxysmal tonic upgaze of infancy has been described as an early phenotypic manifestation of *CACNA1A* pathogenic variants, consistent with the age-dependent expression of channelopathy-related movement disorders [4]. More broadly, age-specific dystonic features, including benign paroxysmal torticollis, are well-recognized in the *CACNA1A* phenotypic continuum, while adulthood may be associated with the emergence of cervical dystonia presenting paroxysmally or as a chronic feature [4].

When episodic ataxia occurs in conjunction with a broader constellation of features, including fluctuating weakness, neurodevelopmental impairment, early-onset paroxysmal movement disorders, or episodic alterations of consciousness, EA2 should be actively considered in the differential diagnosis.

## 4. Conclusions

In conclusion, we described a case of EA2 caused by a de novo *CACNA1A* pathogenic variant that manifested as a broad spectrum of neurological and neurodevelopmental symptoms. The complexity of the patient’s clinical presentation, comprising paroxysmal tonic upward gaze, fluctuating weakness, paroxysmal alterations in consciousness, and persistent cognitive impairment, obscured the underlying etiology and delayed the diagnosis of EA2. This case underscores the shift toward viewing EA2 as part of a larger *CACNA1A*-related disorder continuum. Early genetic diagnosis is essential, as it facilitates the timely implementation of targeted therapies such as acetazolamide and ensures the provision of comprehensive neurodevelopmental support and longitudinal monitoring for this evolving condition.

## Data Availability

The original contributions presented in this study are included in the article. Further inquiries can be directed to the corresponding author.

## Abbreviations

The following abbreviations are used in this manuscript:

EA2	Episodic ataxia type 2
FHM1	Familial hemiplegic migraine type 1
SCA6	Spinocerebellar ataxia type 6
ADHD	Attention-deficit/hyperactivity disorder
DTR	Deep tendon reflex
MRC	Medical Research Council
GBS	Guillain–Barré syndrome
IVIG	Intravenous immunoglobulin
NCS	Nerve conduction studies
EMG	Electromyography
RNS	Repetitive nerve stimulation
NMJ	Neuromuscular junction
